# Inter-Hemispheric Oscillations in Human Sleep

**DOI:** 10.1371/journal.pone.0048660

**Published:** 2012-11-07

**Authors:** Lukas L. Imbach, Esther Werth, Ulf Kallweit, Johannes Sarnthein, Thomas E. Scammell, Christian R. Baumann

**Affiliations:** 1 Department of Neurology, University Hospital Zurich, Zurich, Switzerland; 2 Department of Neurosurgery, University Hospital Zurich, Zurich, Switzerland; 3 Department of Neurology, Beth Israel Deaconess Medical Center, Boston, Massachusetts, United States of America; University Medical Center Groningen UMCG, The Netherlands

## Abstract

Sleep is generally categorized into discrete stages based on characteristic electroencephalogram (EEG) patterns. This traditional approach represents sleep architecture in a static way, but it cannot reflect variations in sleep across time and across the cortex. To investigate these dynamic aspects of sleep, we analyzed sleep recordings in 14 healthy volunteers with a novel, frequency-based EEG analysis. This approach enabled comparison of sleep patterns with low inter-individual variability. We then implemented a new probability dependent, automatic classification of sleep states that agreed closely with conventional manual scoring during consolidated sleep. Furthermore, this analysis revealed a previously unrecognized, interhemispheric oscillation during rapid eye movement (REM) sleep. This quantitative approach provides a new way of examining the dynamic aspects of sleep, shedding new light on the physiology of human sleep.

## Introduction

Sleep is generally considered to be a global and highly symmetrical brain state. Several studies, however, have shown that sleep can exhibit local phenomena, such as electroencephalography (EEG) spectral changes in parts of the cortex in relation to learning [1], [2], [3]. Furthermore, functional MRI and EEG studies have revealed widespread and presumably spontaneous fluctuations in cortical activity during sleep and wakefulness [4], [5], [6], [7], [8]_ENREF_6. These fluctuations show high temporal correlations between distant brain regions and are therefore interpreted as a spontaneously active neural network. During sleep, these resting state networks [9] persist and exhibit slowly (<0.1 Hz) fluctuating activity over time [10], [11], [12]. Considering this local and temporal variability in human sleep, the question arises whether these dynamical aspects may be observed in EEG analysis of healthy consolidated sleep.

By means of behavioral, neurophysiologic and electroencephalographic criteria, sleep may be roughly divided into rapid eye movement (REM) sleep and non-REM (NREM) sleep. NREM sleep may be further subdivided according to the amount of EEG slow wave activity (SWA). Developed in 1969 and adapted in 2007, this classification relies on manual scoring of sleep based on characteristic features of the EEG, electrooculogram, and electromyogram [13], [14] of a human expert. Recently, numerous methods for computer-based automatic sleep stage classification have been proposed using different approaches (e.g. rule-based automatic sleep staging [15], methods based on Bayesian probability [16], or sleep scoring using artificial neural networks [17]). Many classifiers provide an agreement with human scoring that lies in a similar range as the agreement of visual scoring obtained by different experts (approximately 80% agreement [18], [19], [20]). These approaches allow for a standardized analysis of behavioral states, but provide little information on the dynamic spatial and temporal properties of sleep.

Regarding the *spatial* domain, however, it is well established that during consolidated sleep, significant regional variability is observed [21], [22]. As an extreme example, slow wave sleep in marine mammals such as dolphins and fur seals can occur unilaterally, in a pattern that alternates slowly between the left and the right cortical hemispheres [23], [24], [25]. Similarly, in sleep deprived rodents and humans, EEG delta activity during NREM sleep can predominate unilaterally [26], [27]. Some of this regional variation may be a consequence of prior waking activities. For example, a motor learning task can increase SWA in a circumscribed contralateral cortical region [1]. In addition, local populations of cortical neurons of rats may exhibit a sleep-like pattern of activity even while the EEG and behavior indicate wakefulness [3]. Finally, high functional connectivity in different EEG bands has been demonstrated during sleep by analysis of spatial EEG patterns, suggesting an organized underlying neural network with local and long distance connectivity [28], [29]. These studies suggest that during sleep, significant regional EEG variability occurs that may be linked with behavioral changes.

Considering the *temporal* variability of sleep, conventional scoring in 30 second epochs limits analysis of dynamic properties of sleep such as the assessment of changes of sleep behavioral states. For example, conventional scoring presents transitions between behavioral states as if they were instantaneous, though the visual appearance of the EEG suggests the transitions are more gradual with intermediate patterns of activity that defy clear-cut categorization. Similarly, scoring of EEG data in epochs makes it very difficult to examine fluctuations in EEG power over time within a given behavioral state. To overcome these difficulties, various quantitative approaches of EEG analysis have been proposed. Recently, through recurrence analysis of sleep EEG, dynamic markers over time scales of milliseconds have been used as a small scale measure of dynamic brain state activity in different sleep stages [30]. In addition, various model driven approaches focus on the dynamics of the cortical EEG during sleep to describe transitions between distinct sleep stages [31], [32], or to measure and quantify the depth of sleep by spectral analysis techniques [33]. Another promising and novel method of EEG analysis has been introduced in rats [34], [35] and further developed in a mouse model for healthy and pathological sleep [36]. In this approach, behavioral changes are described in a 2-dimensional state space that is derived from spectral characteristics of the EEG. Importantly, by automated spectral analysis of subsequent EEG-epochs, this approach allows for a quantitative and un-biased analysis of the temporal dynamics of sleep in detail.

To gain new perspectives on the physiology of human sleep, we developed an adapted state space analysis technique to study the dynamics of state transitions, regional changes in sleep, and the velocity of transitions between states. We hypothesized that dynamic analysis of healthy sleep, applied in combination with simultaneous measurements in different brain regions, would improve upon the current methods of scoring behavioral states and provide new insights into sleep physiology.

## Materials and Methods

### Participants and EEG Analysis

We analyzed sleep recordings of 14 right-handed, healthy volunteers (6 men, 8 women, 30±8 years old, range: 20–46), who had been screened as healthy controls for a clinical study. All participants reported a regular sleep pattern and had no history of sleep disturbances. Patients with regular alcohol consumption were excluded. BMI >25 kg/m^2^ was accepted, if the polysomnographic findings were normal (see Table S2 for details). The study was approved by the local ethical board (*Kantonale Ethikkommission Zürich*) and all subjects gave written informed consent prior to enrolment. Each subject had overnight polysomnography from 23∶00 to 07∶00, without a prior adaptation night. Six EEG channels were recorded (F3, F4, C3, C4, O1, O2) and referenced to linked bilateral auricular electrodes (A1+A2). All recordings were acquired using Embla N7000 Systems and Somnologica Software (Embla Systems Inc., Broomfield, CO 80021, USA). EEG signals were recorded at a sampling rate of 100 Hz. Manual sleep scoring was performed in non-overlapping 30-s epochs by two experienced sleep researchers (E.W. and U.K.) according to standard criteria [13], [14] for 30****s epochs. In a second step, each epoch was sub-divided into epochs of 5****s length and sleep scoring was adapted if necessary. The adaptation to 5****s epoch length was performed in a systematic way as follows: After the conventional 30-s epoch scoring using the AASM rules to describe the clinical and polysomnographical characteristics of our healthy subjects (Table S2), sleep stages were re-scored in 5-s epochs, with each epoch being assigned a sleep stage comprising the greatest percentage of the epoch. Thus stage W (wake) was determined by the presence of alpha activity and/or rapid eye movements, NREM1 was determined by the presence of low-amplitude mixed-frequency (4–7 Hz) waves and/or vertex sharp waves or slow eye movements. The start of stage NREM2 was defined by the presence of K complexes or sleep spindles. Stage NREM2 continued if low-amplitude, mixed-frequency EEG rhythm was present in epochs that contained or were preceded by K complexes or sleep spindles. Stage NREM2 ended with an arousal or when sleep transitions to stage WAKE, NREM1, NREM3, or REM followed. An epoch was scored as stage NREM3 if at least 50% of the epoch was occupied by slow wave EEG activity. An epoch was considered stage REM if it contained low-amplitude, mixed-frequency EEG activity and low chin EMG tone that was the lowest level in the study and either had rapid eye movements or was preceded by stage REM sleep. REM sleep ended by transition to stage WAKE or any NREM sleep stage as described above. Epochs between definite NREM2 and definite stage REM epoch with a distinct drop in chin EMG, low-amplitude, mixed-frequency EEG activity and absence of K complexes or spindles were scored as stage REM.

This shorter epoch length of 5s (instead of the typically used 30****s epoch length) was chosen for several reasons: First, the shorter time interval enabled to detect rapid changes in the sleep EEG, which allowed for the description of dynamic aspects of sleep. Second, when scoring in 30****s intervals, we observe presumably mixed sleep states, that show characteristics of different sleep stages during short periods of time: These short lasting state transitions could thus be assigned to the corresponding sleep stage (e.g. a short arousal during NREM sleep was scored as a single WAKE epoch of 5****s length). Regarding spectral analysis, this sub-classification of 30****s EEG epochs in 5****s sub-epochs allowed therefore for a higher intra-state consistency of the EEG raw data. Finally, from a more methodological point of view, an interval of 5****s length provides an optimal compromise between a high frequency resolution for spectral analysis (long epoch length) and an adequate temporal resolution (short epoch length) for the dynamic sleep analysis.

Signal processing of the raw data and statistical modelling as described below was performed using MatLab (The MathWorks Inc., Natick, MA, 2009).

### Adaptation of State Space Analysis for Human Sleep

To produce a topographically well-defined state space for analysis of sleep dynamics, we adapted a dimensionality reduction approach. This method was recently introduced for analyzing rodent sleep [34], [36], but to our best knowledge, it has not been used to analyze human sleep. In this approach, multidimensional EEG spectral information in each epoch is reduced to a lower dimensional space by determining ratios of different spectral frequency bands. Each 5-s EEG epoch is thus represented as a single point in a 2-dimensional state space. For appropriately chosen frequency ratios sleep epochs form clusters representing distinct behavioral states. To adapt this technique to human sleep, we used a probability-based optimization of the relevant spectral ratios to determine the most informative frequencies for distinguishing stages of human sleep: First, a Fast Fourier Transformation (FFT) was applied on each 5-s epoch after multiplication by a Hann window to address the problem of edge discontinuities (zero padding was used to expand the signal in each 5-epoch of 500 data points to a window size of 512 points). Second, based on variable frequency bands, the corresponding frequency ratios were determined for each epoch. To filter for short-term fluctuations in the resulting time series, the data was filtered with a running window average (10-point Hann window). Assuming normally distributed ratios within each sleep stage, a 2-dimensional Linear Discriminant Analysis (LDA) was implemented as a discrete classifier function for sleep stages. Finally, we calculated the positive predictive value by comparing the predicted and the manually derived sleep scoring (e.g. a predictive value of 80% corresponds to an agreement with the manual scoring in 80% of all cases). Importantly, the predictive value in this model is dependent on the initially chosen frequency bands.

In a second optimization step, we systematically varied the defining frequency parameters to maximize the predictive value of the derived classification (i.e. to optimize the agreement with manual scoring). To address this high dimensional optimization problem, we first implemented a genetic algorithm [37] (fitness function: LDA classifier; paired regrouping of frequency bands as genetic operator; mutation rate: 10%) to globally search for optimal frequency bands. For this optimization step, the whole data set was used (n = 14). Finally, we used an unconstrained, nonlinear minimization function (Nelder-Mead algorithm; MatLab) for local optimization. Importantly, for any further calculations, these frequency ratios were held fixed and the same ratios were used in the analyses of all individuals and all EEG-data. A detailed mathematical description of this approach is delivered as Supporting information.

### Velocity-dependent Sleep Modelling

Velocity in the state space is defined as the Euclidean distance between two subsequent states divided by the time interval between these states. Rodent studies revealed that high velocities in the state space are an important measure of behavioral state instability [36]. On the other hand, we hypothesized that periods with low velocities represent consolidated, stable sleep. Therefore, we heuristically defined a velocity cut-off to distinguish stable sleep periods (‘slow’) from transitions and fluctuations (‘fast’). To optimize the detection of stable clusters with low intra-state variability, we smoothed the original sleep trajectories with a running window average (50-point wide Hann window). Here, a longer window was chosen, to emphasize state-to-state trajectories with higher velocity, as compared to stable sleep phases. We then used a LDA function to automatically classify the resulting clusters corresponding to WAKE, NREM1, NREM2, NREM3, and REM sleep.

### Autocorrelation of Time-dependent Laterality

To measure differences in state space velocities between the left and right hemispheres (v_left_ and v_right_), we defined the relative laterality score as 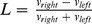 for each 5-s epoch in frontal, central and occipital electrodes. This score potentially fluctuates between -1 and 1 (positive values indicate faster velocity in the right hemisphere; negative values represent faster velocities on the left; and 0 indicates identical velocities in both hemispheres). Here, velocities of the *unsmoothed* sleep trajectories were used to ensure accurate time resolution. The resulting time series was further subdivided into segments of 100 epochs (500 seconds). To detect potential periodic patterns in left/right inter-hemispheric differences, we determined the autocorrelation function (length 100 epochs, lag = 90) and checked for periodicity for each segment. Autocorrelation can be interpreted as the similarity of a signal with itself across time. High autocorrelation values indicate a repeating pattern in the original time series with the same time period. Frequency analysis of the autocorrelation function was done using FFT.

Taken together, we propose a frequency-based sleep model that was optimized for agreement with manual sleep scoring and can therefore be used as an automatic classifier function for sleep stages. Furthermore, as the frequency ratios are uninfluenced by prior knowledge of sleep-related frequency bands this approach allows for an un-biased and quantitative sleep analysis independent of prior human sleep scoring.

## Results

### Behavioral States form Consolidated State Space Clusters

**Figure 1:**
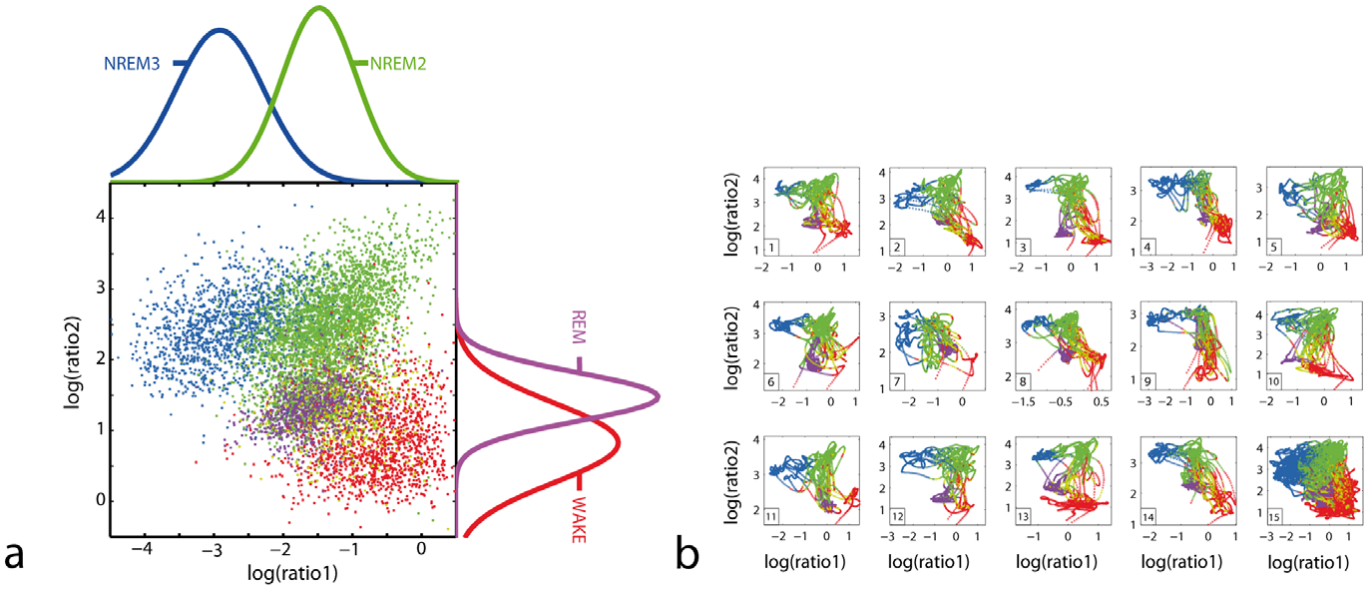
Conserved topography of the sleep state space. (**a**) Summary scatter plot of all sleep states for 14 subjects mapped in a 2-dimensional state space. Each 5****s EEG epoch (raw data) is represented by 2 different frequency ratios plotted on log/log axes. Ratio1 = (8.6 to 19.3 Hz)/(1.0 to 10.9 Hz), Ratio2 = (11.5 to 20.3 Hz)/(17.9 to 31.5 Hz). Colour coding of the clusters is based on expert scoring for WAKE (red), NREM stage 1 (yellow), stage 2 (green), stage 3 (blue), and REM sleep (magenta). Projections of the 2d-probability density distributions are plotted for NREM stage 2 and stage 3 (top edge, ratio1) and WAKE and REM sleep (right edge, ratio2). For better visibility, the figure shows 10% of all data after applying a running window average (6 point-Hann Window) on the raw data to filter for short-term fluctuations. (**b**) Individual sleep trajectories are shown for each subject separately (panel 1–14) and cumulated for all individuals (panel 15, bottom right). Sleep trajectories are smoothed (50 point-Hann Window) for better differentiation of stable (clusters) and transitional sleep states (trajectories). Colour coding is as described in (a).

Calculation of 2 independent frequency ratios in a single EEG electrode (central derivation) allowed for a consistent, probability-dependent automatic classification of behavioral states in all 14 subjects, as illustrated by the projected 1-dimensional probability density estimations in Figure 1a. The topographic arrangement of clusters was conserved across subjects and was independent of age or gender (Figure 1b). Classification of behavioral states based on linear discriminant analysis for trended sleep trajectories resulted in an overall positive predictive value for matching manual scoring of 74%. Analysis of predictive scores according to sleep state showed poor agreement with manual scoring only in transitional sleep states (NREM1), whereas for NREM2 and NREM3 the positive predictive value was ≥80% (Table S1). Importantly, for the automatic classification of behavioural states, the same optimized frequency ratios [Ratio1 = (8.6 to 19.3 Hz)/(1.0 to 10.9 Hz), Ratio2 = (11.5 to 20.3 Hz)/(17.9 to 31.5 Hz)] were used for all individuals without further adaptation.

### Velocity in State Space is a Measure of Behavioral State Instability

**Figure 2:**
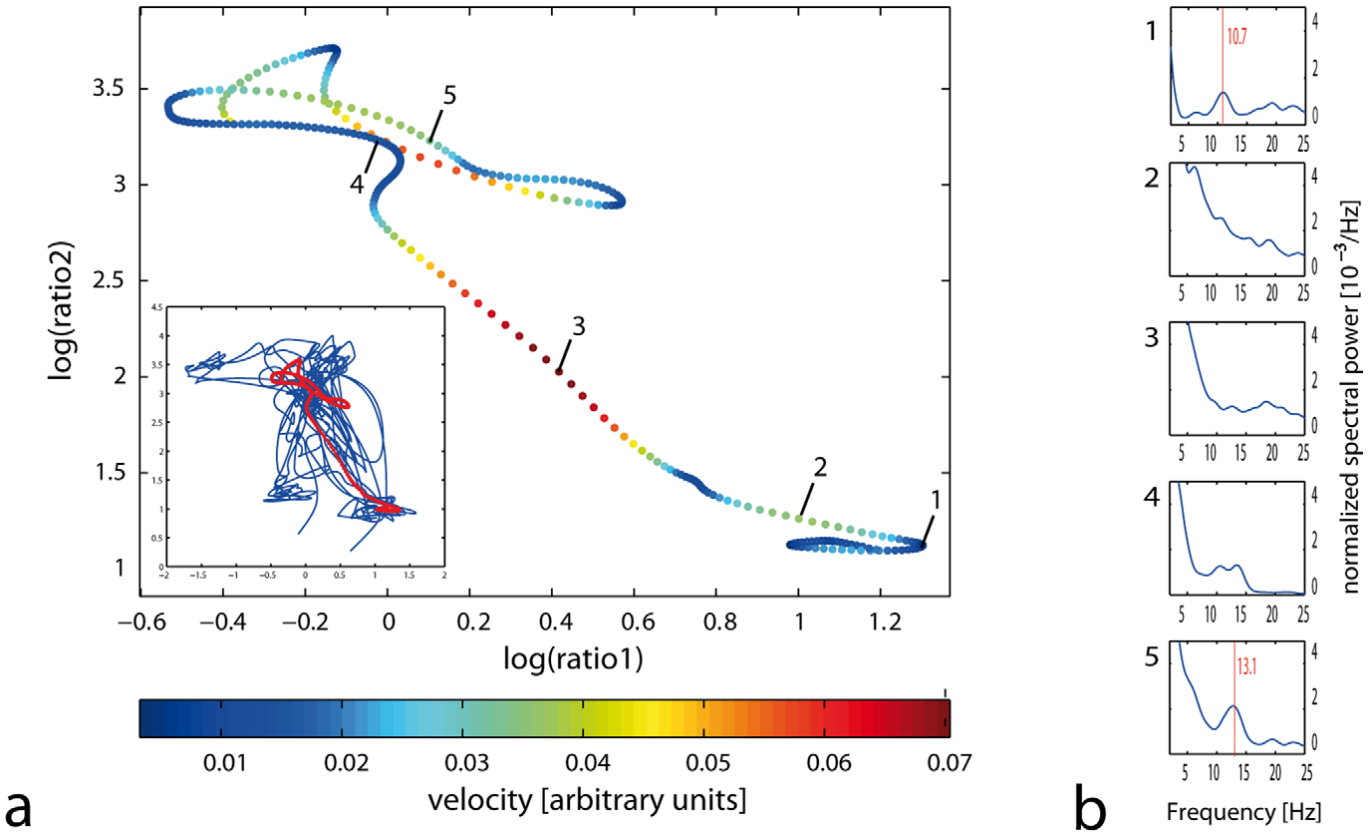
Trajectory for a single WAKE to NREM stage 2 transition. (**a**) Consecutive points in the state space (each representing a 5****s EEG-epoch) during a fast state transition (relevant trajectory (red) as shown in embedded figure) Points are colour coded according to velocity (bottom: colour bar legend: arbitrary velocity units). (**b**) EEG spectral power at different time points during this transition (as indicated by points 1–5 in Figure 2a). Note the uniform and distinctive spectral distribution in slow velocity states. Typical spectral peaks are indicated for point 1 (alpha activity in WAKE: 10.7 Hz) and point 5 (beta activity in NREM2: 13.1 Hz) by a thin red line. During the transitional epochs (points 2–4), the spectra are intermediate.

**Figure 3:**
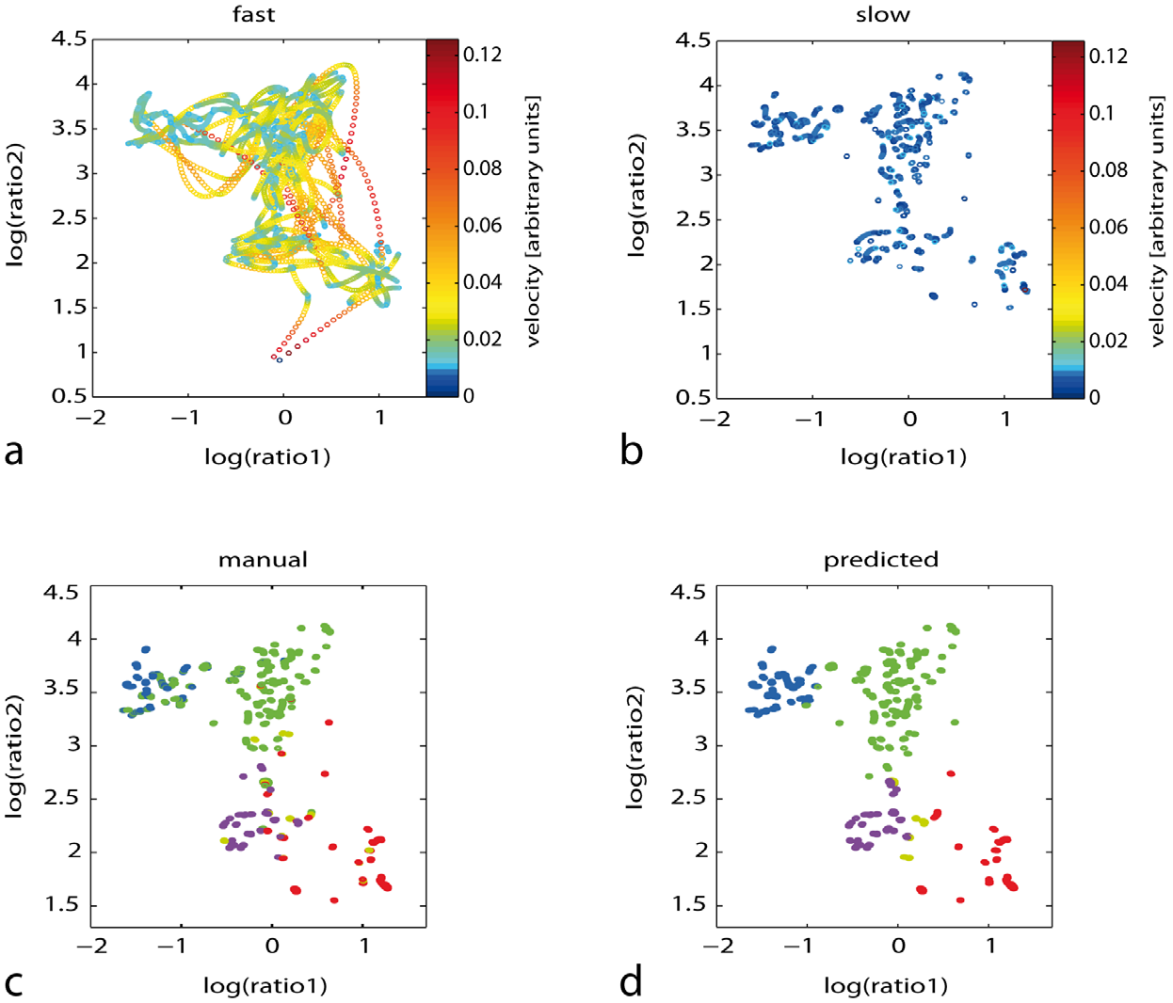
Subdivision of sleep states by velocity. States of sleep were subdivided into high velocity states (i.e. transitions and fluctuations) (**a**), and slow states (i.e. stable clusters) (**b**), by a heuristic velocity limit. Points are colour coded according to velocity (right side: colour bar legend: arbitrary velocity units). Comparison of the manual classification (**c**) with automatic probability based classification (**d**) for a whole night data set for one representative subject is shown. Colour coding: WAKE (red), NREM stage 1 (yellow), stage 2 (green), stage 3 (blue), and REM sleep (magenta).

High velocity states corresponded either to rapid transitions between states or fluctuations within a state, whereas low velocity states formed consolidated clusters. Analyzing sleep trajectories, we found that velocities in state space in 5-s intervals increased abruptly during transitions between behavioral states (Figure 2a). This can be explained partly by a ‘geometrical’ effect, as during state transitions a longer distance in state space (between 2 clusters) has to be covered. However, we also observed high velocity trajectories *within* clusters (Figure 3 a/b). To test whether state space velocity indeed reflects spectral variability, we analyzed the corresponding spectral information at different time points for a given transition: We found typical spectral sleep patterns only in stable (slow) states, whereas during rapid transitions the spectral information is less preserved (Figure 2b). Accordingly, in a sub-analysis of state space velocity with respect to different sleep stages, we found the highest mean velocities in (transitional) sleep stage NREM1, whereas in REM and deep NREM sleep, the mean velocity were significantly lower (Figure S1a). Similarly, comparing transitions between and within clusters (i.e. short trajectories that start and end in the same sleep cluster), the highest velocities occurred with state-to-state transitions (Figure 2). Finally, in agreement with the anterior predominance of sleep EEG, we found a fronto-occipital gradient of velocity in state space for NREM and REM sleep (Figure S1b), indicating more spectral variability in frontal as compared to occipital derivations. Considered together and in agreement with rodent studies [34], [36], these results indicate that low velocities in the state space correspond to consolidated sleep phases with stable EEG spectra over time, and velocity can therefore be interpreted as a quantitative measure of behavioral state instability.

### Consideration of Velocity Improves the Classification of Behavioral States

This approach allowed us furthermore to differentiate between stable and transitional sleep states: To distinguish consolidated, stable periods of sleep from rapid state transitions and fluctuations, we separated epochs into those with low or high velocities. We found well-defined clusters in the low velocity domain (i.e. for stable periods of sleep) for all individuals (Figure 3), whereas fast periods had trajectories arcing between clusters (Figure 1b and Figure 3a). Because the clusters for slow states were better separated, the automatic classifier function based on 2D-probability density was more reliable for *slow* sleep states as compared to *faster* transitional states (Table S1). Automatic categorization in the low velocity domain had a mean positive predictive value of 80% (all subjects) to match manual scoring which is similar to inter-expert variability [18], [19], [20]. By comparison of the automatic scoring performance for slow and fast states separately, we found that – particularly for NREM3 and REM sleep - scoring of slow states resulted in a better agreement with manual scoring, whereas for light sleep states (NREM1, NREM2) we observed a similar agreement in both groups (Table S1).

### Post-hoc Test/train Approach

Because the frequency ratios were determined using the whole data set, there is a possible risk of over-fitting in the proposed model. To test for this possibility, we have confirmed the classification procedure by a post-hoc test/train approach for 2 randomly chosen groups (n = 7 each). The previously determined ratios were left unchanged, but the Linear Discriminant Analysis (LDA) classifier was fitted only on the training group (n = 7) and in a second step the derived discrete classifier function was tested on the ‘naive’ data set (testing group, n = 7) without any information about human scoring. The positive predictive value was then evaluated by comparing the predicted and the manually derived sleep scoring as described above for the testing group only. As compared to the results for all subjects we have found similar values for the testing group (positive predictive value of 70% for all states and 80% for slow sleep states, respectively), indicating that the proposed algorithm operates effectively also on a previously unknown data set (Figure S4).

### State Space Velocity Oscillates Rhythmically between Left and Right Hemisphere

**Figure 4:**
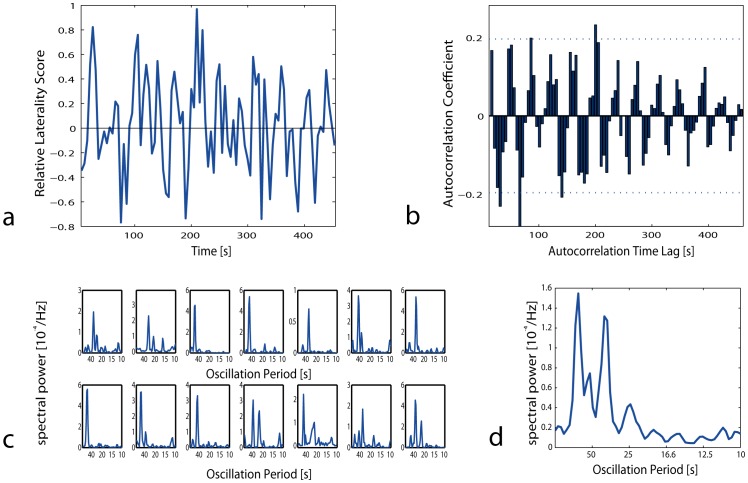
Laterality Analysis of REM sleep. (**a**) Time series data of relative laterality of velocity in corresponding central electrodes (C3 vs C4) for 100 successive REM sleep epochs for one individual. (**b**) Sample autocorrelation of the same period as shown in (a) demonstrating a stable oscillation with a period of about 40****s. Approximate 95% confidence measures for the hypothesis of uncorrelated white noise are indicated by a dotted thin blue horizontal line (p = 0.05, N = 100). (**c**) Fourier analysis (FFT) of REM sleep autocorrelations as in Figure 4b during one selected REM sleep bout for each subject. (**d**) Mean spectral analysis of REM sleep autocorrelation of all 14 subjects.

Next, we examined whether there are regional differences in the stability of sleep across the hemispheres. To measure inter-hemispheric variability, we compared velocities in the left and right hemispheres using corresponding electrodes (e.g. C3 vs. C4). Left/right asymmetry was determined by calculating a relative laterality score for each 5****s epoch. This resulted in a rapidly fluctuating time series representing a constantly changing hemispheric predominance of velocity in state space (Figure S2). During periods of consolidated sleep, state space velocity oscillated rhythmically between the left and right hemispheres (Figure 4). This oscillating pattern was predominantly seen in REM sleep. To examine this oscillation further, we calculated the autocorrelation function for fixed sleep bouts of 500****s length. This approach confirmed the finding of a recurrent pattern of inter-hemispheric oscillations during consolidated sleep phases (Figure 4b).

All 14 subjects had stereotypic oscillating patterns in REM sleep with a mean period of 48 seconds (range: 32–80). This interhemispheric oscillation occurred predominantly in central cortical regions but less in frontal and occipital regions (Figure S3). During NREM sleep and also in WAKE periods, velocities were mostly symmetrical in both hemispheres though shorter interhemispheric oscillations with similar periods occurred in all stages (including WAKE).

Finally, to determine whether this interhemispheric oscillation is an artefact of our method, we tested our data against uncorrelated random velocities and a modelled sinusoid-alternating pattern. As expected, for simulated random velocities with the same distribution as the original data, we found no significant autocorrelations. The modelled sinusoid function, however, showed an alternating pattern very similar to the one observed for the left-right hemispheric oscillations (Figure S2). Furthermore, the oscillating pattern occurred predominantly in central and parietal regions, as shown in the simultaneous analysis of sleep bouts in different electrodes (Figure S3), indicating that an artificial effect in our method seems unlikely, as it would affect all derivations in the same way. These comparative analyses indicate that we observed a genuine, periodically oscillating pattern between both hemispheres.

## Discussion

State space EEG analysis of human sleep generated well-defined patterns of sleep behavioral states with low inter-individual variability. Compared to manually scored sleep stages, the overall positive predictive value for correct automated identification of traditional behavioral states was 74% for all states and 80% for consolidated sleep states, i.e. clusters with low velocities (n = 14 subjects). Contrariwise, we propose that high velocities in state space reflect transitions and represent less stable behavioral states. In addition, this technique revealed an interhemispheric oscillation of behavioral state stability during consolidated REM sleep in humans that to the best of our knowledge has not been described previously.

### Sleep Stage Prediction by State Space Analysis

The presented probability-based sleep analysis revealed a well-defined and relatively uniform topography of the constructed state space for all individuals. This enabled an automatic accurate prediction of sleep states based on frequency ratios of a single EEG channel alone with positive predictive values similar to the variability between examiners using manual scoring based on EEG, electromyography and electrooculography [18], [19], [20]. In contrast to manual scoring however, this approach represents an unbiased algorithm that scores sleep in a reproducible scorer-independent way and allows for a comparable and quantitative sleep EEG analysis. Furthermore, this algorithm can be performed on a single EEG-channel and therefore allows for comparison of quantitative sleep measures between different EEG derivations on a 5****s time scale. Although our approach is fully dependent on the underlying spectral information, our classification algorithm works particularly well for REM sleep (Table S1). This is a rather surprising finding, considering that based on spectral information alone differentiation of REM sleep is generally less reliable, whereas e.g. deep sleep (NREM3) can be separated from other sleep stages by calculation of delta spectral power (0–4 Hz). This advantage of our approach might be explained by an intrinsic reduction of spectral variability in the algorithm by calculating frequency *ratios* of normalized EEG-spectra.

Transitional states, however, are a challenge with manual and automated scoring because their EEG spectral characteristics are ill-defined and change rapidly. Description of a state to state transition by conventional scoring rules can result in significant ambiguity. For some clinical and research applications, it may be better to divide sleep into consolidated, stable states and transitional fluctuating states on the other hand. With stable states that vary little over time, a clear-cut classification into different behavioral states is feasible and the underlying spectral similarity is well conserved. This fits our observation that automated scoring using state space clusters works best for the sub-population of slow sleep states. On the other hand, transitional states might be better represented by dynamic quantitative sleep parameters such as state-to-state trajectories or velocities in state space rather than conventional sleep scoring. Future projects could apply these concepts to obtain quantitative and comparable data on healthy and pathological sleep, fully independent from manual sleep scoring.

### Inter-hemispheric Oscillation

Based on this finding, we examined whether instability is a local or a global phenomenon. In other words: can humans produce consolidated, stable sleep in one hemisphere while simultaneously producing a less stable state in the other hemisphere?

In some species (e.g. marine mammals [23], [24], birds [38] and possibly certain reptiles [39]), alternating unilateral slow-wave sleep is the most frequently encountered sleep pattern [40]. Unihemispheric sleep offers an elegant solution for the conflicting needs for sleep and vigilance and has been linked with possible functional aspects of sleep in these animals such as predator detection by enhanced unihemispheric arousability [41] or uninterrupted breathing during sleep in marine mammals [25]. Although long-term local changes of slow wave sleep occur in rats and humans [1], [42], [43], land mammals apparently are not able to produce a similar alternating sleep pattern [40]. Considering both the distinct lateralization of the human brain and the suggested complex functional aspects of sleep (e.g. memory consolidation), it would be rather surprising for sleep in humans to consist of fully homogeneous and symmetrical brain states. Our finding of an oscillation of sleep EEG dynamics between the left and right hemisphere during consolidated REM sleep reveals that healthy humans are in fact able to produce an intermittent rhythm of alternating hemispheric predominance. Simply put, during consolidated REM sleep, one hemisphere may be in a slow velocity, stable state, whereas the other hemisphere is in a faster, less stable state. This rhythmic pattern fluctuates between the hemispheres approximately every minute. This alternation is suggestive of the left-right alternating sleep pattern in slow wave sleep of dolphins and other sea mammals, but in humans, this interhemispheric oscillation has a much shorter period and is less obvious when using standard EEG analyses. Furthermore, the alternating pattern emerges mainly during stable periods of REM sleep lasting for about 10 minutes.

A well known intermittent EEG marker of unstable sleep is the cyclic alternating pattern (CAP), which consists of long-lasting periodic activity of two alternating electroencephalogram patters [44], [45]. Although we claim that velocity in state space is an indirect measure for sleep instability, we believe that our finding of interhemispheric oscillations in state space is not directly related to CAP for several reasons: CAPs occur primarily in NREM sleep, whereas the observed interhemispheric sleep oscillation is predominant in REM sleep. Furthermore, the time period of EEG changes in CAP is typically shorter as in our case. In addition, the EEG changes in CAP have a symmetrical distribution among both hemispheres. Finally and most importantly, by visual inspection of our EEG raw data we do not observe any alternating stereotyped EEG patterns or any correlation with periodic movements, respiratory fluctuations or tonic/phasic REM sleep periods during sleep phases in which the inter-hemispheric oscillation occurred. Furthermore, saw-tooth waves or other sleep EEG patterns are generally symmetrically over both hemispheres and do generally not show an intermittent inter-hemispheric pattern and are therefore unlikely to be linked with this oscillation.

### Methodological Aspects and Possible Limitations

Many important aspects of sleep can be studied using conventional EEG analysis techniques, but novel approaches are needed to examine intermediate states and changes in sleep across time in a quantitative way. Here, we propose velocity in state space as a novel measure of sleep instability. This interpretation is based on the finding that during transitional states and fluctuations state space velocity rises significantly, because the underlying spectral information varies rapidly. Here, the question arises, whether this variability might simply reflect spectral noise or intermittent EEG artefacts. We believe this is rather unlikely for several reasons. By taking the *ratio* of different spectral power bands for each sleep epoch, the noise level of the EEG spectra is actually diminished for all epochs equally. Furthermore, noise levels could not explain the exclusively higher velocities for state transitions (as seen in Figure 3 a/b), as noise would be expected to occur randomly during all states in a similar amount. Finally, velocity differed according to sleep stage and according to EEG derivation in all individuals in the same way (Figure S1). By the same argument as above, this is very unlikely to be related to the noise or artefact level in the EEG. We therefore conclude that high velocities in state space indeed correlate with less stable sleep.

In a similar way, we have to consider that frequency *ratios* are used in this model: When using ratios of parameters that change rapidly over time, one has to consider the risk of over-emphasizing or concealing temporal variability when analyzing a time series (e.g. small changes of the denominator may lead to enormous changes in the corresponding ratio). For this reason, we have worked with a logged ratio approach: Taking the log of a ratio converts the division into a subtraction (because mathematically log(a/b) = log(a)-log(b)). Small changes of either the denominator or the numerator are therefore represented indeed by small changes in the state space (on a logarithmical scale).

Furthermore, we have to consider the possibility that the slow oscillating pattern of velocity that we have observed might be an artefact of the rather complex analysis method. This seems rather unlikely for several reasons. Regarding the predominant occurrence in REM sleep and the strictly rhythmic inter-hemispheric alteration, muscle artefacts or signals generated by the electrodes are unlikely to cause a rhythmically recurring oscillating pattern with a constant frequency. Furthermore, as seen in Figure S3, we have observed this pattern predominantly in central derivations, whereas an artificially generated pattern would have been expected to occur in all derivations equally.

As an additional limitation, all volunteers were recorded during their first night in the sleep lab without any prior adaptation. Although the first night effect is known to influence the occurrence of different sleep stages, it seems rather unlikely to affect the findings of the inter-hemispheric oscillation that occurred during consolidated REM sleep. However, an influence of the first night effect on our findings cannot be ruled out.

Regarding the shortened epoch length, one might argue that changing the interval length from 5****s would also change the observed oscillations. At this point, it cannot be ruled out completely, that changing the underlying analysis method would also result in a different inter-hemispheric pattern (possibly in other sleep stages). However, we are confident that our method provides a sufficient resolution for detecting the presented slow oscillation: Analyzing the data in 5****s intervals corresponds to a sampling rate of 0.2 Hz, which allows for detecting oscillations with frequencies up to ∼0.1 Hz. The observed oscillations are at approximately 0.02 Hz (50****s Time-period) and lie therefore well within the spectral range of this technique. However, the occurrence of faster inter-hemispheric oscillations or more local EEG variations cannot be ruled out because of limited temporal (5****s interval) and spatial (8 electrode montage) resolution in the current setting. Finally, we have to address the question of consistency between the scoring with shortened epoch length as compared to the traditional 30s-scoring. To validate the described adapted scoring rules (see Material/Methods), the independently derived sleep scores (5****s vs. 30****s) have been compared for all volunteers and showed a high level of overall agreement (85%, see Figure S5a/b/d). Importantly, for the remaining 15% of epochs that showed no agreement with the traditional 30****s, we suggest that because of the higher temporal resolution of the 5****s scoring these states might be more accurately described by the 5****s epoch length. Put differently, we argue that in fact the 30****s scoring fails to capture short fluctuations in the sleep EEG in up to 15% of all epochs. This argument is supported by the fact, that for a similar percentage of all scored 30****s epoch, we have scored 2, 3 or even 4 different behavioral states in the 5****s scoring (Figure S5c).

### Possible Relevance of the Inter-hemispheric Oscillation

For now, the function of this oscillation remains unknown, but its predominant occurrence during REM sleep suggests a relation to REM sleep function. Much evidence has shown that REM sleep is critical for the development of the brain and for procedural learning [46], [47] by induction of hippocampal long-term potentiation [48], and NREM sleep is possibly linked to neuronal recuperation and the enhancement of declarative memories [49]. Accordingly, in recent theoretical and experimental approaches it has been suggested that during sleep neural networks exhibit a high amount of local connectivity, which might be optimal for information processing in complex systems [29], [50]. In this context, the observed oscillating rhythm may hypothetically be explained by an inter-hemispheric neural crosstalk during a memory consolidation process in REM sleep. Alternatively, the oscillating REM sleep rhythm might be related to an alternating pattern of enhanced arousability as in birds or sea mammals. This could be linked to the observation that in humans auditory evoked potentials during sleep show a variable left/right asymmetry [51] suggesting a greater receptiveness for auditory input in the corresponding hemisphere [40].

Though the function of this interhemispheric rhythm remains uncertain, the fact that all subjects had REM sleep oscillations with similar time periods suggests that this finding represents a genuine aspect of human sleep physiology that may reflect dynamic interactions between the hemispheres during REM sleep.

## Supporting Information

Figure S1 **Distribution of state space velocity with respect to sleep behavioral state and brain region. (a)** Mean velocities showed a characteristic distribution with highest velocities in NREM1 for all 14 subjects. Average over all individuals demonstrated significantly higher velocities in NREM1 as compared to NREM2, NREM3 and REM sleep (* = p<0.01, paired t-test, n = 14). Error bars indicate SEM (n = 14). **(b)** Velocity in state space showed a fronto-occipital gradient for all subjects with highest velocities in frontal and central electrodes and lower velocities in occipital derivations for NREM (filled blue) and REM sleep (white). Average over all individuals are shown (Error bars indicate SEM, n = 14).
(TIF)

Figure S2 **Comparison of laterality time series with random models. (a)** ***Time series raw data:*** Histogram plot of relative laterality showed a symmetrical distribution over time (left panel), while time series raw data fluctuated rapidly between left and right hemispheres (middle panel, 200 REM sleep epochs, one subject). Sample autocorrelation of the same period with respect to autocorrelation time lag showed an oscillating pattern (right panel). **(b)** ***Random model:*** For comparison, the same analysis was performed on Rayleigh-distributed random numbers with a distribution of laterality (left panel) and a corresponding rapidly fluctuating time series (middle panel) similar to real data (a). However, autocorrelation of the random data shows no oscillating pattern (right panel) **(c)** ***Sinusoid model:*** Histogram (left panel), time series (middle panel) and autocorrelation (right panel) for a sinus function underlying uniformly distributed random noise simulated the rhythmically oscillating pattern as observed in REM sleep (compare to right panel in (a)). Dotted blue lines represent approximate 95% confidence measures, as described in Figure 4.
(TIF)

Figure S3 **Simultaneous measurements of interhemispheric oscillation in different brain regions.** The laterality score of velocity was calculated in corresponding electrodes for *frontal* (F3 vs F4), *central* (C3 vs C4), *parietal* (P3 vs P4) and *occipital* (O1 vs O2) derivations. The analysis was performed for the same 100 successive REM sleep epochs simultaneously in each electrode pair. The typical oscillating pattern was only seen in central and parietal brain regions, whereas in frontal and occipital derivation no oscillating pattern could be observed (upper panels). Frequency analysis for the oscillating pattern (FFT of REM sleep autocorrelations) confirmed the predominant slow oscillation in central and parietal electrodes, but no definable frequency peak in frontal and occipital regions was observed (lower panels).
(TIF)

Figure S4 **Test/Train Approach for 2 randomly selected subgroups (2x n = 7).** Left panel: Summary scatter plot of all sleep states for 7 subjects that were used for *training* the LDA algorithm. Colors represent *manual* scoring. Right panel: State space scatter plot of all sleep states for the other 7 subjects that were used for *testing* of the classifier. Epochs were scored by the automatic classifier without prior knowledge of the manual scoring. Colors represent automatic scoring. Agreement with manual scoring was 70% for all states and 80% for slow sleep states. Color-coding is the same as in Figure 1.
(TIF)

Figure S5 **Comparison of 30 s- and 5 s-scoring. (a/b) Sleep hypnograms 5 s/30 s for 1 representative volunteer.** Scoring of behavioural states was performed for 30****s epochs **(a)** and 5****s epochs **(b)** showing a similar sleep structure with a higher variability in the 5****s scoring. **(c) Number of different 5 s behavioural states per 30 s epoch for all n = 14 subjects**. For each 30****s epoch the number of different behavioural states (1–4 bs) in the 6 corresponding 5****s epochs was determined and shown for each traditional behavioral state separately. **(d) Validation of 5 s scoring as compared to the 30 s scoring epoch for all n = 14 subjects**. Comparison of the 5s-scoring with the traditional 30s-scoring showed a high level of agreement in all behavioural states (WAKE, NREM1, NREM2, NREM3, REM) and overall agreement of 85% (OVERALL).
(TIF)

Table S1 **Relative abundance of sleep stage and positive predictive values for** ***slow*** **and** ***fast*** **states in all individuals (n = 14). (a)** Considering all epochs, we find an overall agreement with manual scoring in 74% (ALL). Differentiation of high velocity and low velocity states resulted in an increased positive predictive value for automatic classification of slow sleep stages (SLOW), whereas fast states are less reliably predicted (FAST). This effect was predominantly observed in consolidated deep sleep (NREM3, REM), whereas for the transitional sleep stage NREM1 a poor performance of the automatic classification was observed. **(b)** Comparison of manual and automated classification of behavioral states by a confusion matrix. Numbers indicate fractions of correctly assigned sleep stages, when comparing manual (y-axis) with automated scoring (x-axis) for both fast (left panel) and slow (right panel) sleep states for all volunteers (n = 14). Color-coding refers to the fractions as shown on the matrix (percentage values).
(TIF)

Table S2 **Clinical characteristics and polysomnographical findings of all included volunteers (n = 14).** Abbreviations: gender (g), Epworth Sleepiness Scale (ESS), Body Mass Index (BMI), Sleep Efficiency (Seff), Sleep Latency to S2 (SL[S2]), Rem Sleep Latency (RL), Periodic Limb Movements (PLM), Apnea-Hypopnea-Index (AHI), Total Time in Bed (TIB), Total Sleep Time (TST), Total sleep Time from sleep onset (SPT), Relative occurrence of stage NREM1 (S1), NREM2 (S2), Deep Sleep (S3+S4), REM Sleep (REM) and Wake (Wake).
(TIF)

MatLab m-file S1 (score_p10): **MatLab function for automatic scoring of sleep EEG-spectra by linear discriminant analysis**. Requires MatLab mat-files 1–3: *mu_sigma.mat, P_si.mat, xmat.mat* (See file header for instructions).
(M) Click here for additional data file.

MatLab m-file S2 (oplot2d_50): **MatLab function for plotting the sleep spectrum** ***spec*** **on a 2d state space** (See file header for instructions). MatLab mat-files containing all parameters for automatic sleep scoring.
(M) Click here for additional data file.

MatLab mat-file S1 MatLab mat-files containing all parameters for automatic sleep scoring.
(M) Click here for additional data file.

MatLab mat-file S2 MatLab mat-files containing all parameters for automatic sleep scoring.
(M) Click here for additional data file.

MatLab mat-file S3 MatLab mat-files containing all parameters for automatic sleep scoring.
(M) Click here for additional data file.

Mathematical description S1 Provides a detailed mathematical description of the optimization algorithm for the derived state space model.
(DOCX)
