# Supplementary figures and images for: Inter-Hemispheric Oscillations in Human Sleep
Source: PLoS One. 2012 Nov 7;7(11):e48660. doi: 10.1371/journal.pone.0048660 (PMC3492490; doi:10.1371/journal.pone.0048660)

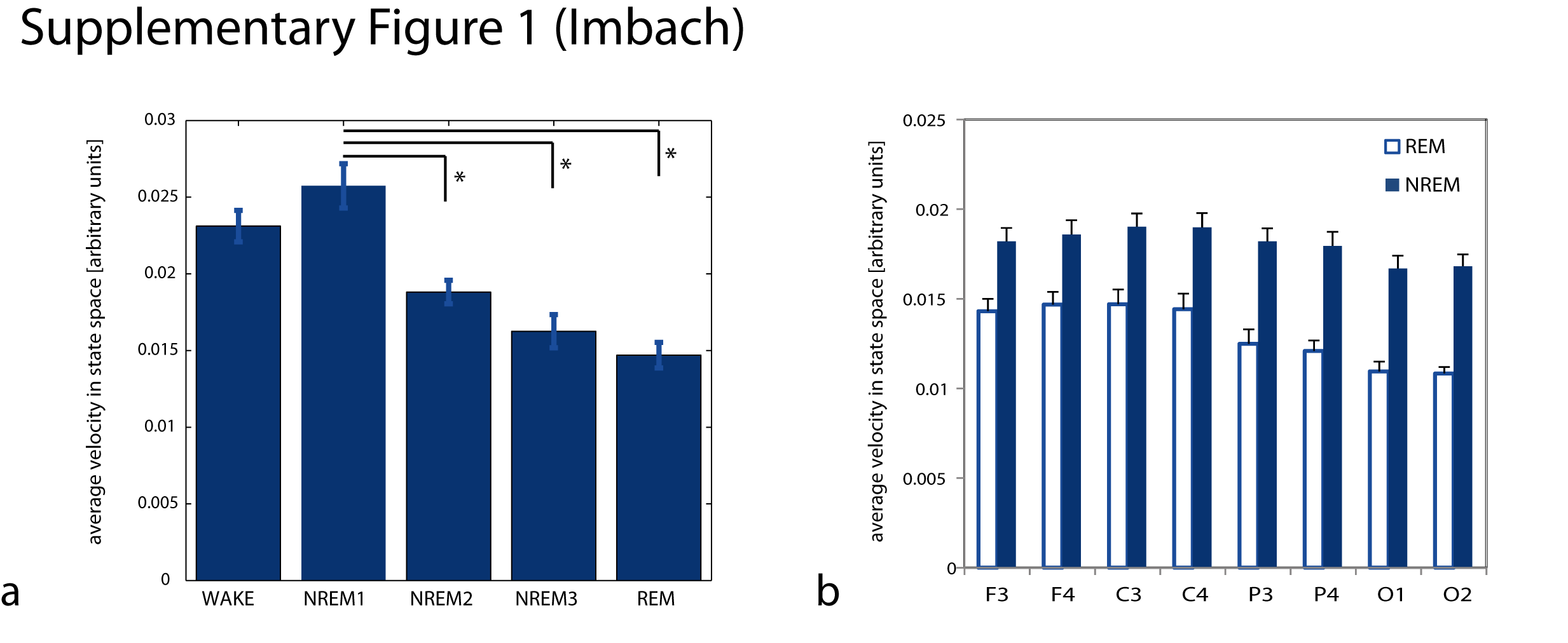

Supplement: Figure S1 — Distribution of state space velocity with respect to sleep behavioral state and brain region. (a) Mean velocities showed a characteristic distribution with highest velocities in NREM1 for all 14 subjects. Average over all individuals demonstrated significantly higher velocities in NREM1 as compared to NREM2, NREM3 and REM sleep (* = p<0.01, paired t-test, n = 14). Error bars indicate SEM (n = 14). (b) Velocity in state space showed a fronto-occipital gradient for all subjects with highest velocities in frontal and central electrodes and lower velocities in occipital derivations for NREM (filled blue) and REM sleep (white). Average over all individuals are shown (Error bars indicate SEM, n = 14). (TIF) [file pone.0048660.s001.tif]

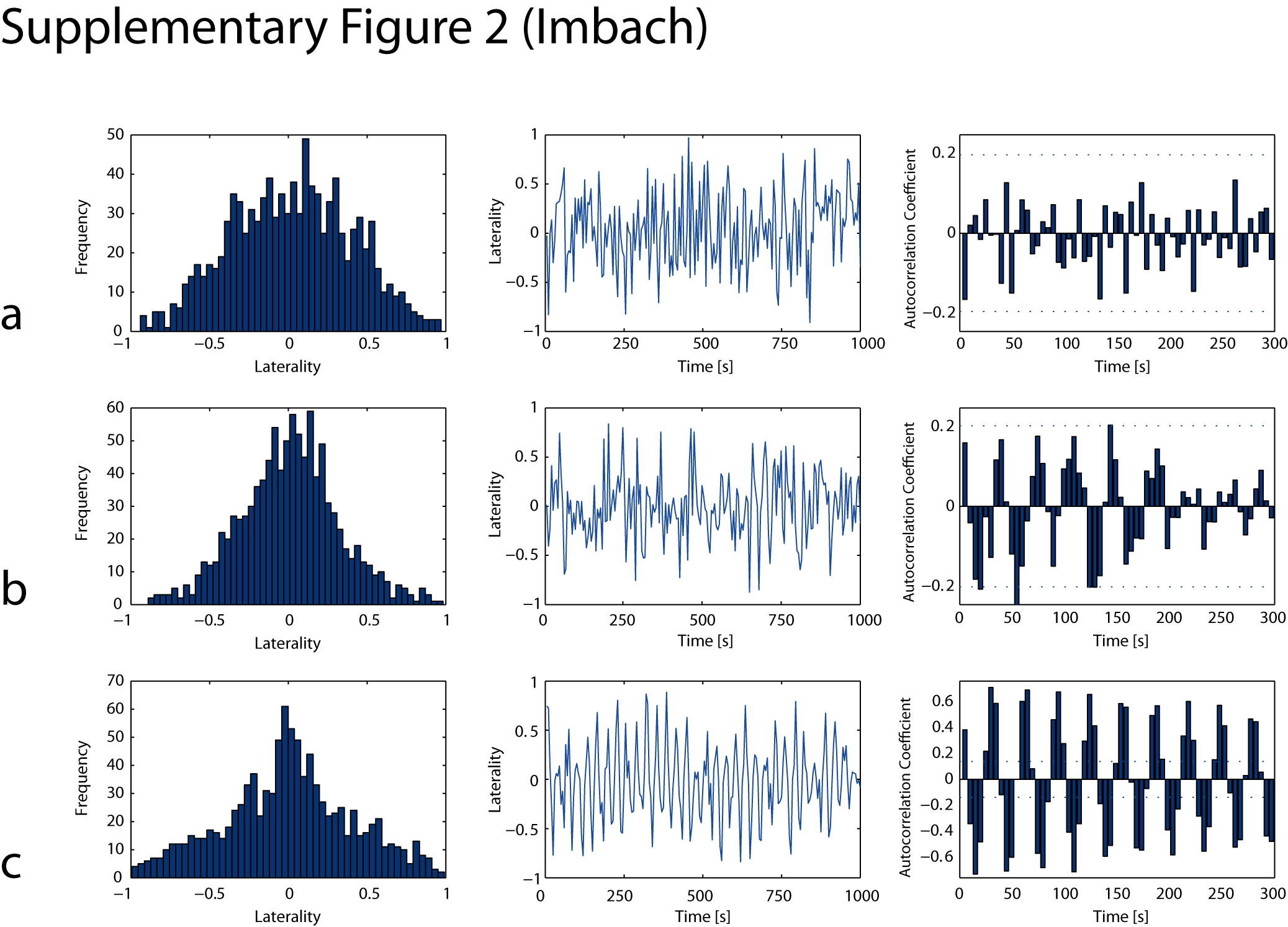

Supplement: Figure S2 — Comparison of laterality time series with random models. (a) Time series raw data: Histogram plot of relative laterality showed a symmetrical distribution over time (left panel), while time series raw data fluctuated rapidly between left and right hemispheres (middle panel, 200 REM sleep epochs, one subject). Sample autocorrelation of the same period with respect to autocorrelation time lag showed an oscillating pattern (right panel). (b) Random model: For comparison, the same analysis was performed on Rayleigh-distributed random numbers with a distribution of laterality (left panel) and a corresponding rapidly fluctuating time series (middle panel) similar to real data (a). However, autocorrelation of the random data shows no oscillating pattern (right panel) (c) Sinusoid model: Histogram (left panel), time series (middle panel) and autocorrelation (right panel) for a sinus function underlying uniformly distributed random noise simulated the rhythmically oscillating pattern as observed in REM sleep (compare to right panel in (a)). Dotted blue lines represent approximate 95% confidence measures, as described in Figure 4. (TIF) [file pone.0048660.s002.tif]

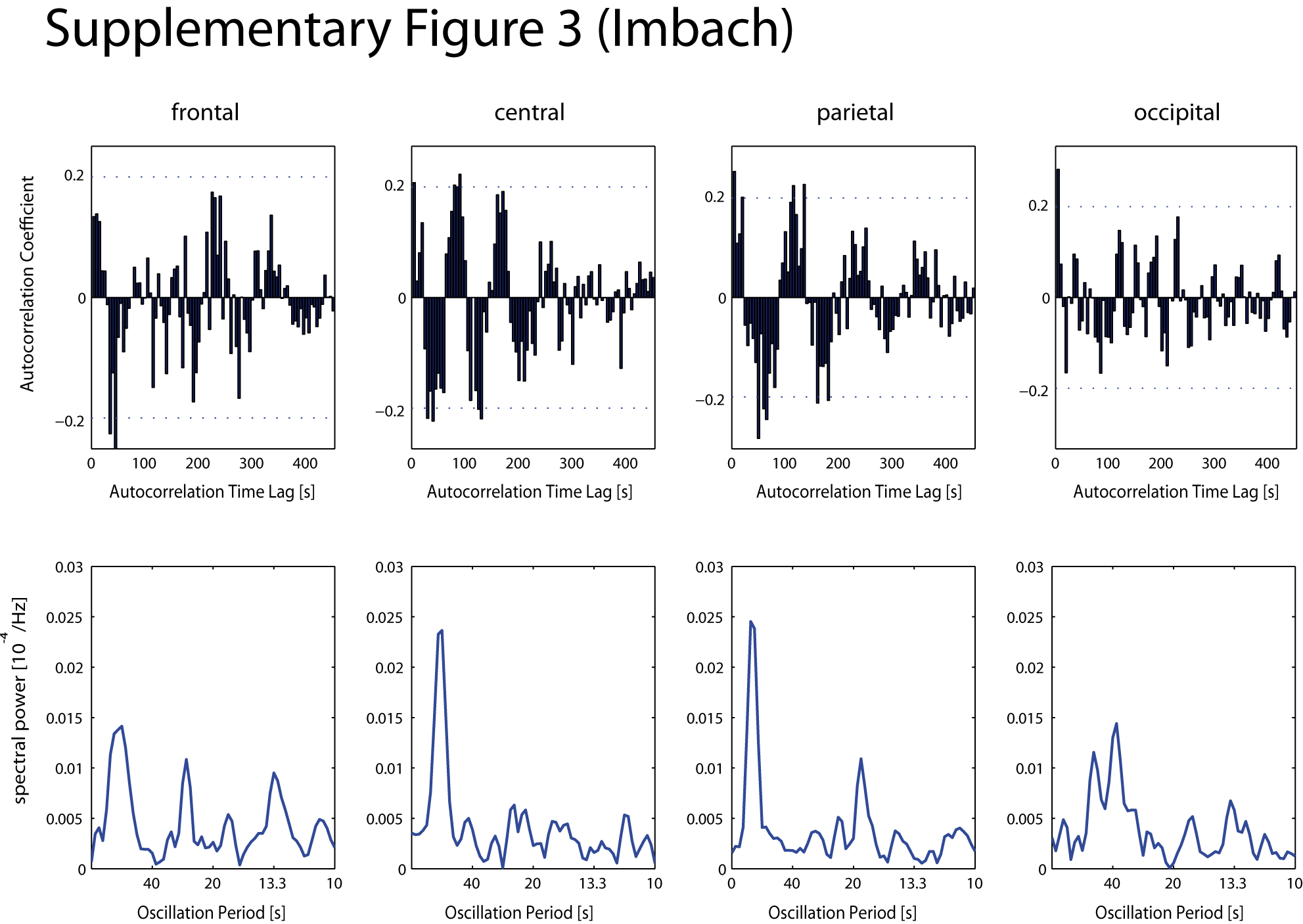

Supplement: Figure S3 — Simultaneous measurements of interhemispheric oscillation in different brain regions. The laterality score of velocity was calculated in corresponding electrodes for frontal (F3 vs F4), central (C3 vs C4), parietal (P3 vs P4) and occipital (O1 vs O2) derivations. The analysis was performed for the same 100 successive REM sleep epochs simultaneously in each electrode pair. The typical oscillating pattern was only seen in central and parietal brain regions, whereas in frontal and occipital derivation no oscillating pattern could be observed (upper panels). Frequency analysis for the oscillating pattern (FFT of REM sleep autocorrelations) confirmed the predominant slow oscillation in central and parietal electrodes, but no definable frequency peak in frontal and occipital regions was observed (lower panels). (TIF) [file pone.0048660.s003.tif]

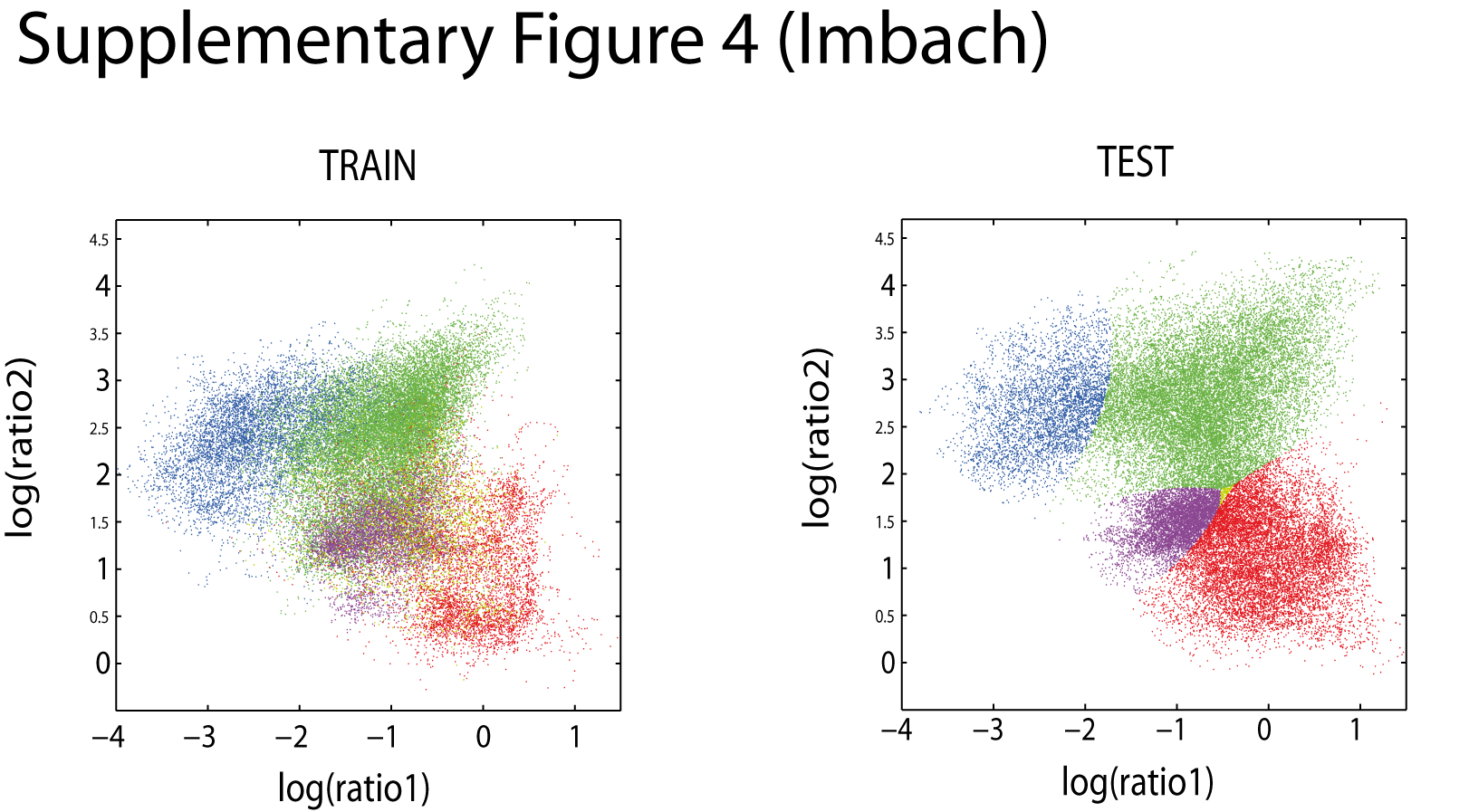

Supplement: Figure S4 — Test/Train Approach for 2 randomly selected subgroups (2x n = 7). Left panel: Summary scatter plot of all sleep states for 7 subjects that were used for training the LDA algorithm. Colors represent manual scoring. Right panel: State space scatter plot of all sleep states for the other 7 subjects that were used for testing of the classifier. Epochs were scored by the automatic classifier without prior knowledge of the manual scoring. Colors represent automatic scoring. Agreement with manual scoring was 70% for all states and 80% for slow sleep states. Color-coding is the same as in Figure 1. (TIF) [file pone.0048660.s004.tif]

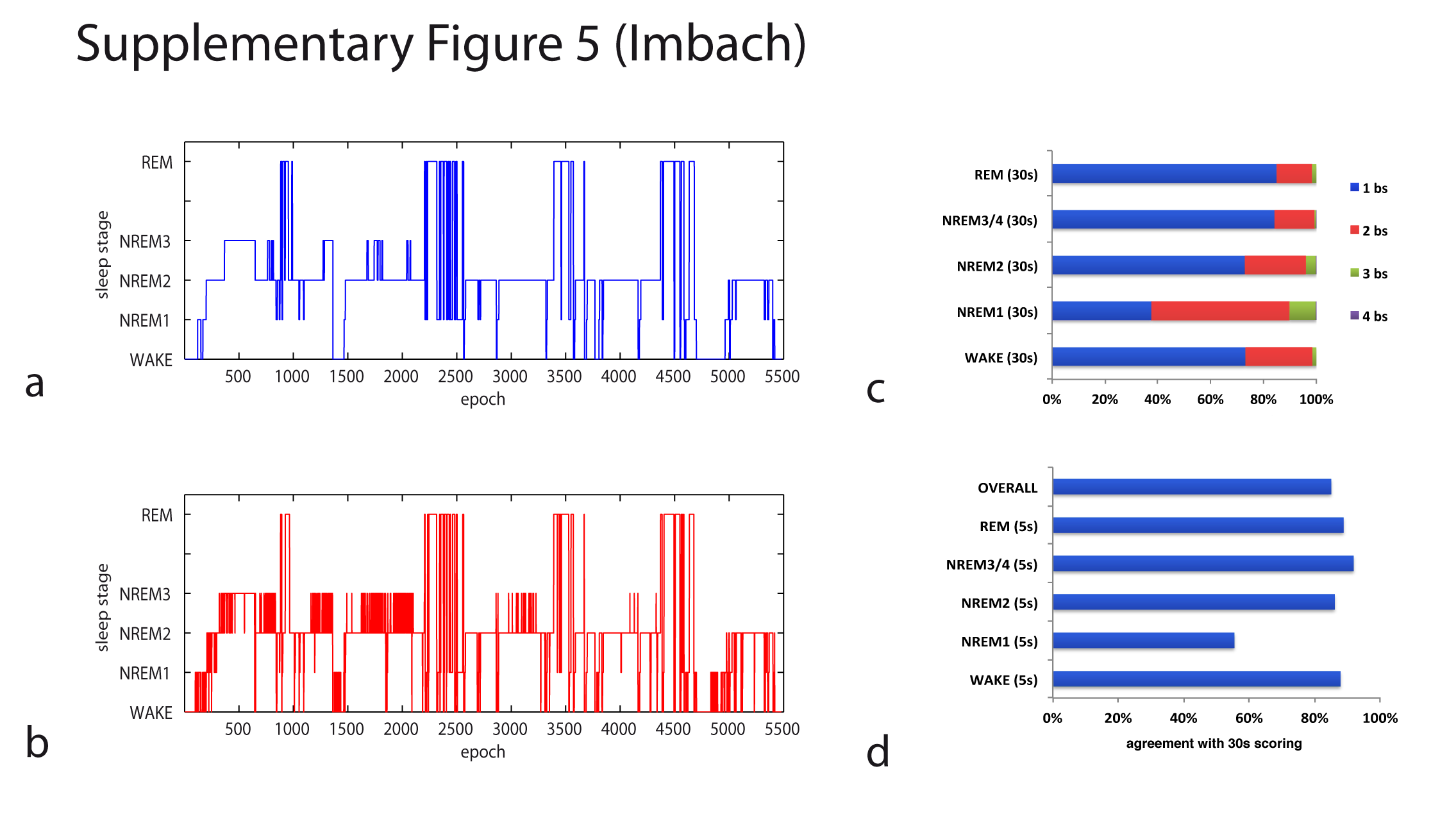

Supplement: Figure S5 — Comparison of 30 s- and 5 s-scoring. (a/b) Sleep hypnograms 5 s/30 s for 1 representative volunteer. Scoring of behavioural states was performed for 30s epochs (a) and 5s epochs (b) showing a similar sleep structure with a higher variability in the 5s scoring. (c) Number of different 5 s behavioural states per 30 s epoch for all n = 14 subjects. For each 30s epoch the number of different behavioural states (1–4 bs) in the 6 corresponding 5s epochs was determined and shown for each traditional behavioral state separately. (d) Validation of 5 s scoring as compared to the 30 s scoring epoch for all n = 14 subjects. Comparison of the 5s-scoring with the traditional 30s-scoring showed a high level of agreement in all behavioural states (WAKE, NREM1, NREM2, NREM3, REM) and overall agreement of 85% (OVERALL). (TIF) [file pone.0048660.s005.tif]

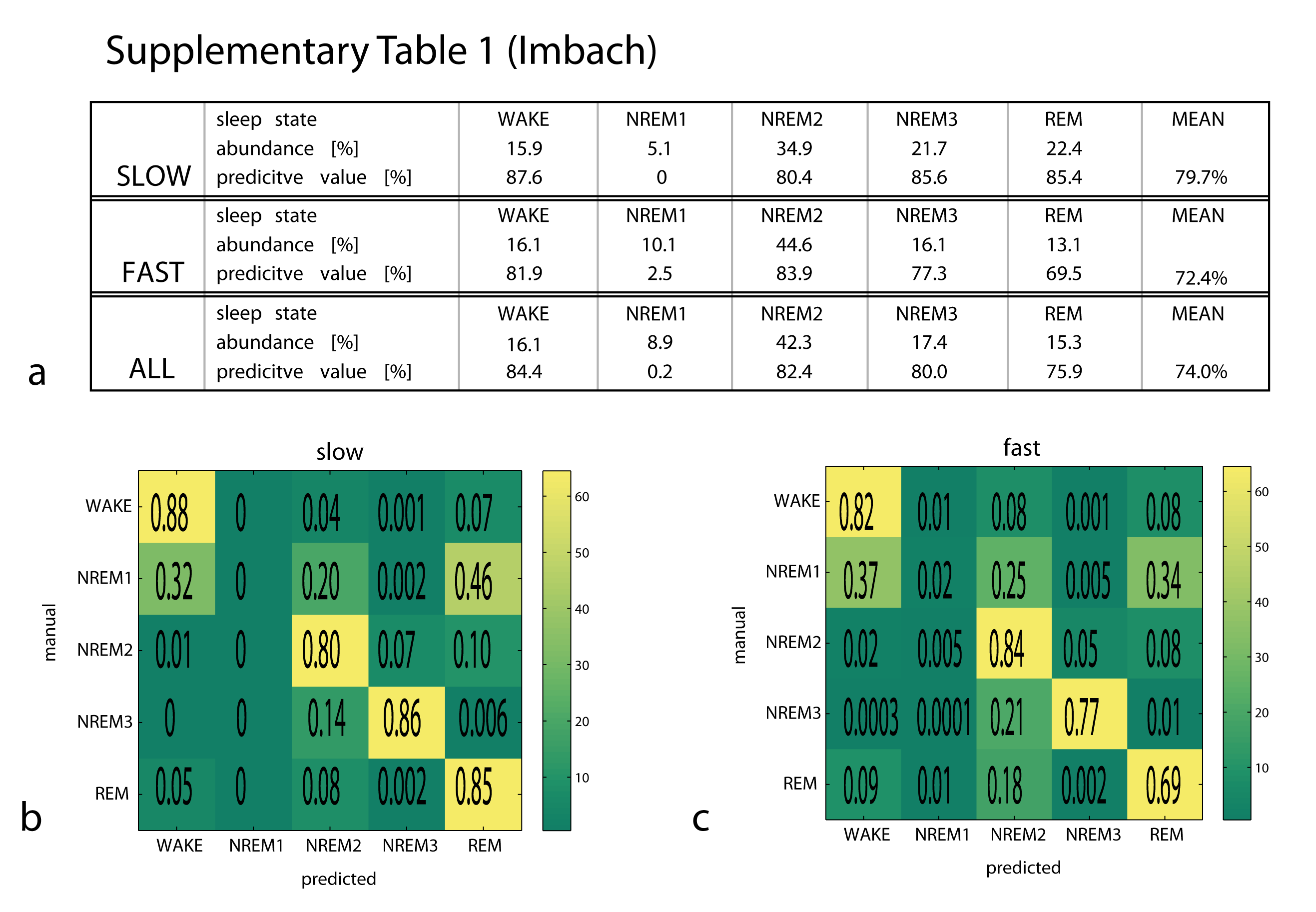

Supplement: Table S1 — Relative abundance of sleep stage and positive predictive values for slow and fast states in all individuals (n = 14). (a) Considering all epochs, we find an overall agreement with manual scoring in 74% (ALL). Differentiation of high velocity and low velocity states resulted in an increased positive predictive value for automatic classification of slow sleep stages (SLOW), whereas fast states are less reliably predicted (FAST). This effect was predominantly observed in consolidated deep sleep (NREM3, REM), whereas for the transitional sleep stage NREM1 a poor performance of the automatic classification was observed. (b) Comparison of manual and automated classification of behavioral states by a confusion matrix. Numbers indicate fractions of correctly assigned sleep stages, when comparing manual (y-axis) with automated scoring (x-axis) for both fast (left panel) and slow (right panel) sleep states for all volunteers (n = 14). Color-coding refers to the fractions as shown on the matrix (percentage values). (TIF) [file pone.0048660.s006.tif]

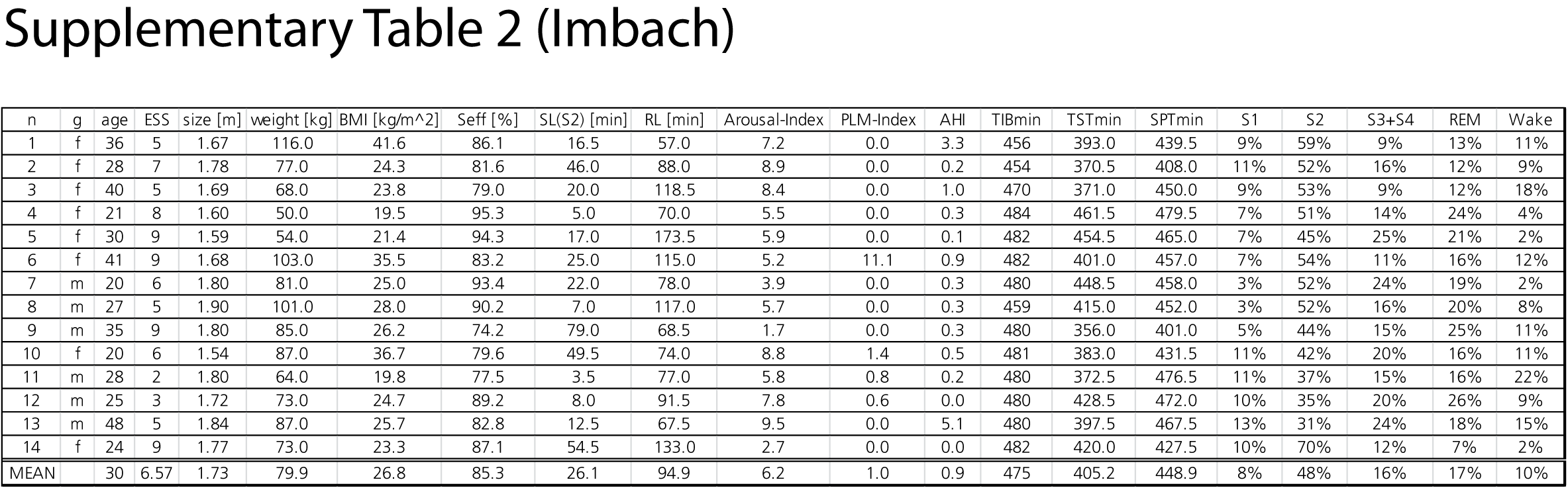

Supplement: Table S2 — Clinical characteristics and polysomnographical findings of all included volunteers (n = 14). Abbreviations: gender (g), Epworth Sleepiness Scale (ESS), Body Mass Index (BMI), Sleep Efficiency (Seff), Sleep Latency to S2 (SL[S2]), Rem Sleep Latency (RL), Periodic Limb Movements (PLM), Apnea-Hypopnea-Index (AHI), Total Time in Bed (TIB), Total Sleep Time (TST), Total sleep Time from sleep onset (SPT), Relative occurrence of stage NREM1 (S1), NREM2 (S2), Deep Sleep (S3+S4), REM Sleep (REM) and Wake (Wake). (TIF) [file pone.0048660.s007.tif]
