# Supplementary material for: Inter-Hemispheric Oscillations in Human Sleep
Source: PLoS One. 2012 Nov 7;7(11):e48660. doi: 10.1371/journal.pone.0048660 (PMC3492490; doi:10.1371/journal.pone.0048660)
Supplement: Mathematical description S1 — Provides a detailed mathematical description of the optimization algorithm for the derived state space model. (DOCX) [file pone.0048660.s013.docx]

**Mathematical description of the state space model**

For the probability-dependent sleep modelling we have implemented a linear discriminant analysis (LDA) with a classifier based on an estimated normal distribution function following a standard approach [[52](#_ENREF_52)]. In general, when used for statistical classification, LDA achieves to make a classification decision by a classifier that relies on a linear combination of its characteristics. For our model we propose the following approach:

Consider a time series of m observations of n-dimensional vectors: $\boldsymbol{s}_{\boldsymbol{i}}$ (i=1…m), where each vector $\boldsymbol{s}_{\boldsymbol{i}}$ represents the spectral information of the i-th EEG epoch (window size of 512 points). Now, for dimensionality reduction, we define new variables as frequency ratios by parameters $q_{j}$ (j=1…8) with $q_{j}\in\left[ 1\ldots512 \right]$. For the i-th observation we introduce a vector **x** with 2 components by:

$${x_{(i)}}^{1}=\frac{\sum_{j=q_{1}}^{q_{2}} s_{ij}}{\sum_{{j=q}_{3}}^{q_{4}} s_{ij}} and {x_{(i)}}^{2}=\frac{\sum_{j=q_{5}}^{q_{6}} s_{ij}}{\sum_{{j=q}_{7}}^{q_{8}} s_{ij}}$$

Each epoch is now characterized by the values of the variables **x**_i_ and its membership to a specific class $k$. In our case we have to consider 2 dimensional data (2 ratios) and k=5 classes (wake, nrem1-3, rem). For a classification of given point **x**_o_ to a specific class ω_o_ we then have to estimate the conditional probability, that an observation belongs to class ω_o_ = k given the value of **x**_o_. Using a Bayesian approach this probability can be written as:

$$P(\omega_{o}=k│x_{o})=\frac{p_{k}*P\left( x_{o} | \omega_{o}=k \right)}{\sum_{j} p_{j}*P\left( x_{o} | \omega_{o}=j \right)}$$

with p_k_ being the a priori probabilities of each class. The conditional probability $P\left( x_{o} | \omega_{o}=k \right)$ can then be estimated by the 2 dimensional normal distribution f_k_ for each class.

$$P(\omega_{o}=k│x_{o})=\frac{p_{k}*f_{k}(x_{o})}{\sum_{j} p_{j}*f_{j}(x_{o})}$$

with the multivariate normal distribution function f_k_**:**

$$\boldsymbol{f}_{\boldsymbol{k}}\boldsymbol{(x,\mu,}\boldsymbol{\Lambda}\boldsymbol{)=}\frac{\boldsymbol{1}}{\sqrt{\boldsymbol{\Lambda}{\boldsymbol{(2}\boldsymbol{\pi}\boldsymbol{)}}^{\boldsymbol{2}}}}\boldsymbol{e}^{\boldsymbol{-}\frac{\boldsymbol{1}}{\boldsymbol{2}}\boldsymbol{(x-\mu)}\boldsymbol{\Lambda}^{\boldsymbol{-1}}\boldsymbol{(x-\mu)'}}$$

Importantly, the covariance matrix $\boldsymbol{\Lambda}$ and the mean values$\boldsymbol{\mu}$ are implicitly dependent on the initially chosen parameters $q_{j}$ (j=1…8) that define the state space model. Therefore, we may write:

$$P(\omega_{o}=k│x_{o})=\frac{p_{k}*f_{k}(x_{o},\boldsymbol{q})}{\sum_{j} p_{j}*f_{j}(x_{o},\boldsymbol{q})}$$

Now, as a simple classification rule we assign each observation to the class with the highest posterior probability (Bayes’ Classification):

$$\left\langle\omega_{o} \right\rangle=\arg\max_{k} \left( P(\omega_{o}=k│x_{o}) \right)=\arg\max_{k} \left( \frac{p_{k}*f_{k}(x_{o},\boldsymbol{q})}{\sum_{j} p_{j}*f_{j}(x_{o},\boldsymbol{q})} \right)$$

Because the denominator is constant for all classes, this simplifies to:

$\left\langle\omega_{o} \right\rangle=\arg\max_{k} \left( p_{k}*f_{k}(x_{o},\boldsymbol{q}) \right)$

Where $\left\langle\omega_{o} \right\rangle$ is the estimate for the classification of an observation with variables **x**_o_.

For a given data set $\boldsymbol{s}_{\boldsymbol{i}}$ (i=1…m), we are now able to calculate the predictive value of this classification as compared to manual scoring. For m observations, we define a function that estimates the predictive value $pv\left( \boldsymbol{q,s} \right)$ by:

$pv\left( \boldsymbol{q,s} \right):=\frac{\sum_{i} \text{δ}(x_{i})}{m}$

with $\delta\left( x_{i} \right)=\left\{ \begin{aligned} 1, if&\arg\max_{k} \left( p_{k}*f_{k}\left( x_{i},\boldsymbol{q} \right) \right)= \omega_{manual} (TRUE) \\ 0, if&\arg\max_{k} \left( p_{k}*f_{k}\left( x_{i},\boldsymbol{q} \right) \right)\neq\omega_{manual} (FALSE) \end{aligned} \right.$

Because the predictive value is now dependent on the 8 dimensional variable $\boldsymbol{q}$, for the optimization of our model we finally have to maximize this function with respect to the model-defining parameters $\boldsymbol{q}$. The optimized frequency parameters $q_{max}$ are therefore defined by:

$$q_{max}=\arg\max_{q} pv(\boldsymbol{q,s}) for \boldsymbol{q}\in\mathbb{R}^{8}$$
